# Dicer structure and function: conserved and evolving features

**DOI:** 10.15252/embr.202357215

**Published:** 2023-06-13

**Authors:** David Zapletal, Karel Kubicek, Petr Svoboda, Richard Stefl

**Affiliations:** ^1^ CEITEC‐Central European Institute of Technology Masaryk University Brno Czech Republic; ^2^ Faculty of Science, National Centre for Biomolecular Research Masaryk University Brno Czech Republic; ^3^ Institute of Molecular Genetics of the Czech Academy of Sciences, v.v.i. Prague 4 Czech Republic

**Keywords:** Dicer, dsRBD, helicase, miRNA, siRNA, RNA Biology, Structural Biology

## Abstract

RNase III Dicer produces small RNAs guiding sequence‐specific regulations, with important biological roles in eukaryotes. Major Dicer‐dependent mechanisms are RNA interference (RNAi) and microRNA (miRNA) pathways, which employ distinct types of small RNAs. Small interfering RNAs (siRNAs) for RNAi are produced by Dicer from long double‐stranded RNA (dsRNA) as a pool of different small RNAs. In contrast, miRNAs have specific sequences because they are precisely cleaved out from small hairpin precursors. Some Dicer homologs efficiently generate both, siRNAs and miRNAs, while others are adapted for biogenesis of one small RNA type. Here, we review the wealth of recent structural analyses of animal and plant Dicers, which have revealed how different domains and their adaptations contribute to substrate recognition and cleavage in different organisms and pathways. These data imply that siRNA generation was Dicer's ancestral role and that miRNA biogenesis relies on derived features. While the key element of functional divergence is a RIG‐I‐like helicase domain, Dicer‐mediated small RNA biogenesis also documents the impressive functional versatility of the dsRNA‐binding domain.

## Graphical abstract

This review discusses recent data on animal and plant Dicer substrate recognition and cleavage in small RNA pathways and highlights the helicase and dsRNA‐binding domains as key elements of functional divergence.

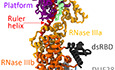

## Introduction

**Figure 1:**
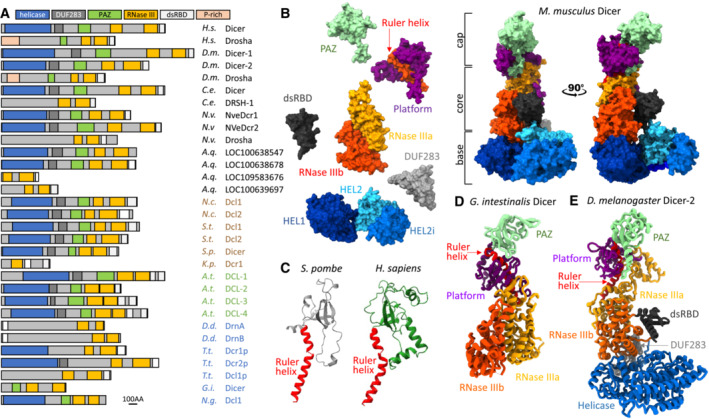
Dicer architecture (A) Domain organization of selected Dicer and Dicer‐like proteins across eukaryotic kingdoms. Animal proteins are in a black font, fungi in brown, plants in green, and Protista in blue. Abbreviations of species names: *H.s.—Homo sapiens*, *D.m.—Drosophila melanogaster*, *C.e.—Caenorhabditis elegans*, *N.v.—Nematostella vectensis* (sea anemone), *A.q.—Amphimedon queenslandica* (sponge), *N.c.—Neurospora crassa* (red bread mold), *S.t.—Sporotrichum thermophile* (thermophilic fungus), *S.p.—Schizosaccharomyces pombe* (fission yeast), *K.p.—Kluyveromyces polysporus*, *A.t.—Arabidopsis thaliana*, *D.d.—Dictyostelium discoideum* (slime mold), *T.t.—Tetrahymena thermophila*, *G.i.—Giardia intestinalis*, and *N.g.—Naegleria gruberi*. Schemes were built based on domain annotations in Genbank, conserved domain search, sequence alignments, and inspection of AlphaFold predictions (Varadi *et al*, 2022). (B) Exploded and normal views of mammalian Dicer [PDB ID: 7YZ4] architecture depicting the key structural modules (Zapletal *et al*, 2022). (C) Comparison of the folds of the PAZ domain of *S. pombe* (gray fold) and *H. sapiens* (green fold). The red connector helix was retained in the fold to indicate orientation of the fold relative to the rest of Dicer. *S. pombe* fold is based on AlphaFold (Varadi *et al*, 2022), for the human Dicer a published structure [PDB ID: 5ZAM] was used (Liu *et al*, 2018). (D) A ribbon model of Dicer of *Giardia intestinalis* based on its crystal structure [PDB ID: 2FFL] (MacRae *et al*, 2006). This Dicer represents a simpler variant lacking the base and the C‐terminal dsRBD. (E) A ribbon model of the full architecture of Dicer‐2 of *Drosophila melanogaster* [PDB ID: 7W0B] (Su *et al*, 2022).

Small RNAs (20–30 nucleotides [nt] long) loaded on Argonaute proteins function as sequence‐specific guides in numerous RNA silencing pathways (reviewed in Ketting, 2011). Small RNAs in many RNA silencing pathways are generated by RNase III Dicer from substrates with double‐stranded RNA structures (reviewed in Jaskiewicz & Filipowicz, 2008). Dicers and numbers of their paralogs in each species vary. Some organisms use a single Dicer for small RNA production, while others have two or more Dicers with distinct features. For example, mammals and *Caenorhabditis elegans* utilize a single Dicer, *Drosophila* two, and *Arabidopsis* has four (Fig 1A). This review summarizes recent remarkable progress on understanding the structural and functional variability of Dicer proteins in different model systems and discusses the main principles of Dicer protein function and evolution.

## Dicer Architecture

Dicer is a general name for RNase III endoribonucleases producing small RNAs for RNA silencing pathways. There is a common order of specific modules present in Dicer across eukaryotic kingdoms, which reflects the common order of protein domains in the primary protein sequence. This protein organization constitutes the canonical Dicer architecture, despite some eukaryotic Dicers differing considerably from it (Fig 1A and B). The three‐dimensional architecture of canonical Dicer consists of three major structural regions: the cap (head), the core (body), and the base (Lau *et al*, 2009; Wang *et al*, 2009; Taylor *et al*, 2013; Liu *et al*, 2018; Fig 1B).

### The core—an internal dimer of the RNase III domains

The rigid core of Dicer‐related enzymes (Fig 1B) is formed by an intramolecular dimer of two RNase III domains, which resembles a bacterial RNase III dimer (Zhang *et al*, 2004; MacRae *et al*, 2006). Each RNase III domain of Dicer cleaves one strand of the substrate (also known as “dicing”). The same intramolecular RNase III dimer organization is common for proteins called Dicer or Dicer‐like, even if they otherwise deviate from the canonical architecture. The RNase III core module is also present in Drosha, a Dicer‐related enzyme (Fig 1A), which likely evolved from a Dicer duplication in a Metazoan ancestor (Maxwell *et al*, 2012; Brate *et al*, 2018). Drosha supports animal miRNA biogenesis as the catalytic core of a nuclear Microprocessor complex that cleaves primary microRNA transcripts (pri‐miRNA) into precursor miRNA (pre‐miRNA) substrates for Dicer (reviewed in Bartel, 2018).

### The cap—the PAZ domain and platform with a connector helix

The cap module is composed of the Piwi/Argonaute/Zwille (PAZ) domain, the platform, and the connector helix (Fig 1B). The PAZ domain is an RNA‐binding domain found in Dicer and Argonaute proteins (Lingel *et al*, 2004; Ma *et al*, 2004). In Dicer, PAZ is a dsRNA‐binding element (Ma *et al*, 2004; MacRae *et al*, 2006) that anchors one end of the substrate. The PAZ domain is present in animal and plant Dicers as well as in Dicer of *Giardia intestinalis* (MacRae *et al*, 2006), a flagellated unicellular parasite. The low sequence conservation of the PAZ domain impedes its recognition by sequence homology analysis with Dicers from the fungi *Schizosaccharomyces pombe* and *Neurospora crassa* (Colmenares *et al*, 2007; Vetukuri *et al*, 2011; Paturi & Deshmukh, 2021). However, despite the lack of sequence conservation, a PAZ‐like fold appears in the structural prediction for Dicer from *S. pombe* (Fig 1C), suggesting that the canonical Dicer architecture evolved in a common ancestor of the main eukaryotic kingdoms.

The PAZ/platform domains in different Dicers may contribute to discriminating Dicer substrates. While the PAZ has specificity for the two nucleotide 3′ overhang (3′ pocket; Lingel *et al*, 2004; Ma *et al*, 2004), the adjacent platform domain may carry a phosphate‐binding pocket, enabling combined binding of both strands of a substrate RNA duplex with the 3′ two nucleotide overhang and assuring efficient and accurate substrate processing (Park *et al*, 2011; Tian *et al*, 2014). The 5′ binding pocket is present in *Drosophila* Dicers and human Dicer but not in *Giardia* or *S. pombe* Dicer. Functional studies suggested that simultaneous anchoring of the 3′ and 5′ ends of the substrate terminus is a feature important for fidelity of small RNA biogenesis (Park *et al*, 2011; Fukunaga *et al*, 2014; Kandasamy & Fukunaga, 2016). A unique phosphate‐binding pocket exists in *Drosophila* Dicer‐2, which is required to bind the 5′ monophosphate of short dsRNA substrates but not long dsRNAs (Cenik *et al*, 2011; Fukunaga *et al*, 2014). Inorganic phosphate binding by the pocket suppresses miRNA processing *in vivo* and alters substrate specificity in favor of long dsRNAs (Fukunaga *et al*, 2014). At the same time, the phosphate‐binding pocket of Dicer‐2 supports length fidelity of siRNA production from long dsRNA (Kandasamy & Fukunaga, 2016). In monocot plants, divergence of the PAZ domain in DCL3 and DCL5 paralogs determines distinct substrate specificity in the biogenesis of distinct classes of 24‐nt siRNAs (Chen *et al*, 2022).

The PAZ domain is key to the biogenesis of small RNAs of defined length by different Dicers, as the length of the product is defined by the distance between the end of the substrate anchored in the PAZ domain and the catalytic center (MacRae *et al*, 2006, 2007). This distance is defined by an α helix (connector helix or ruler helix; Fig 1B, D and E), a structural component first reported in the Dicer structure from *Giardia* (MacRae *et al*, 2006), which directly connects the PAZ domain with the RNase III domains (Fig 1D).

### The base—the N‐terminal helicase domain

The base of the enzyme consists of an N‐terminal helicase domain of the RIG‐I‐like receptor subgroup, which forms a clamp‐like structure near RNase III (Lau *et al*, 2009, 2012; Taylor *et al*, 2013; Liu *et al*, 2018). This domain is composed of three globular subdomains: an N‐terminal DExD/H domain (HEL1), an insertion domain (HEL2i), and a helicase superfamily C‐terminal domain (HEL2). As we will discuss later, the N‐terminal helicase is another structural element involved in substrate recognition and functional differentiation of Dicer homologs as shown by structural and functional analyses (Welker *et al*, 2011; Liu *et al*, 2012; Kidwell *et al*, 2014; Sinha *et al*, 2015; Wang *et al*, 2021; Wei *et al*, 2021; Jouravleva *et al*, 2022; Su *et al*, 2022; Yamaguchi *et al*, 2022; Zapletal *et al*, 2022; Aderounmu *et al*, 2023). It also mediates interactions with regulatory proteins (reviewed in Hansen *et al*, 2019).

### dsRBD and DUF283 domains

Two additional domains are part of the common Dicer architecture: the C‐terminal dsRNA‐binding domain (dsRBD), which localizes near the catalytic core, and the DUF283 domain localized next to the helicase domain at the boundary between the core and the base (Fig 1). DUF283 has a dsRBD fold (Dlakic, 2006) and, like dsRBD, appears to interact with the substrate during dicing (Wang *et al*, 2021; Wei *et al*, 2021; Jouravleva *et al*, 2022; Su *et al*, 2022; Yamaguchi *et al*, 2022; Zapletal *et al*, 2022). The C‐terminal dsRBD has also been implicated in affecting the nucleocytoplasmic localization of Dicer (Vagin *et al*, 2009; Doyle *et al*, 2013).

While the above‐described canonical Dicer architecture (Fig 1B) exists across eukaryotic kingdoms, there are Dicer variants deviating from the cap‐core‐base architecture. The abovementioned Dicer from *Giardia* lacks the base as well as the C‐terminal dsRBD, representing a “minimal Dicer” composed of the core and head parts. There are other Dicers, whose architecture deviates even more. For example, DrnA/B in *Dictyostelium* retains the tandem RNase III core module, the dsRBD is placed at the N terminus and the central part of the protein does not show sequence homology with canonically built Dicers (Hinas *et al*, 2007; Kruse *et al*, 2016; Liao *et al*, 2018; Fig 1A). How DrnA/B determine the length of the product remains unclear. Similarly, abovementioned Drosha proteins may be considered highly derived Dicer variants retaining the core module with the dsRBD (Kwon *et al*, 2016). Remarkably, noncanonical Dicer *Dcr1* in *Kluyveromyces polysporus* carrying just a single RNase III domain and a tandem dsRBD (Fig 1A) is also able to generate small RNAs of specific lengths (Weinberg *et al*, 2011). Structural analyses of *Dcr1* suggested that the siRNA length of ~ 23 nt is generated upon cooperative binding of long dsRNA by an array of Dicers, where dicing by two adjacent Dicers yields a sized siRNA duplex.

## Main Dicer Binding Partners

Dicer interacts with many proteins but two binding partner families stand out: (I) Aproteins from the Argonaute family, which bind small RNAs generated by Dicer and (II) dsRNA‐binding proteins with tandemly arrayed dsRBD, which facilitate substrate recognition, dicing fidelity, and Argonaute loading.

Argonaute proteins form the core of effector complexes and provide another layer for divergence of RNA silencing pathways (reviewed in Meister, 2013). Argonautes exist in both, prokaryotes and eukaryotes suggesting they emerged before the canonical Dicer architecture was formed. Eukaryotic Argonautes have a stereotypical bilobed architecture consisting of four domains: N‐terminal, PAZ, MID and PIWI. The Argonaute PAZ domain binds the 3′ end of the loaded small RNA (Lingel *et al*, 2003; Song *et al*, 2003; Yan *et al*, 2003). The 5′ end of the loaded small RNA is recognized by the MID domain (Boland *et al*, 2010; Frank *et al*, 2010). The PIWI domain has an RNaseH fold and provides nuclease activity for Argonaute homologs able to cleave RNAs complementary to the loaded small RNA (Liu *et al*, 2004; Meister *et al*, 2004). Interaction of human Dicer with AGO Argonaute subfamily was proposed through a region near the RNase IIIa domain (Sasaki & Shimizu, 2007).

The second type of common Dicer interaction partners is dsRNA‐binding proteins with three dsRBDs, such RDE‐4 in *C. elegans* (Tabara *et al*, 1999, 2002). Loquacious (LOQS) and R2D2 in *Drosophila* (Liu *et al*, 2003; Forstemann *et al*, 2005; Jiang *et al*, 2005; Hartig *et al*, 2009; Zhou *et al*, 2009), TARBP2 and PACT in mammals (Chendrimada *et al*, 2005; Haase *et al*, 2005; Lee *et al*, 2006) or double‐stranded RNA‐binding (DRB) family proteins in plants (Clavel *et al*, 2016). These proteins harbor a C‐terminal Type B dsRBD, which exhibits no interaction with RNA, but mediates protein–protein interaction. Interaction with Dicer can be exemplified by mammalian TARBP2 whose C‐terminal type B dsRBD (so‐called MEDIPAL domain) is degenerated and interacts with Dicer's HEL2i region (Laraki *et al*, 2008; Daniels *et al*, 2009; Wilson *et al*, 2015). However, despite their similar architectures, Dicer‐binding dsRBD proteins adapted different roles in substrate recognition and processing in RNA silencing in different species (Liu *et al*, 2003; Chendrimada *et al*, 2005; Haase *et al*, 2005; Parker *et al*, 2006, 2008; Marques *et al*, 2010; Hartig & Forstemann, 2011).

## Dicer Substrates

**Figure 2:**
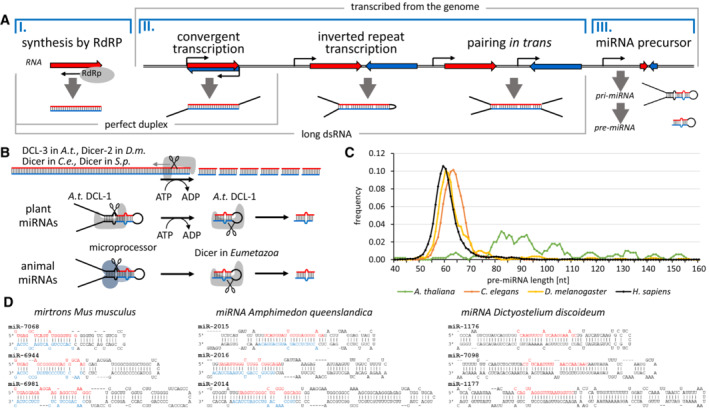
Dicer substrates and principles of their dicing (A) Schematic representation of origins of different dsRNA substrates and their key features. Genomic transcripts producing dsRNA can originate from repetitive transcripts in pathways protecting genome integrity, or from genic sequences in pathways regulating gene expression. (B) Two modes of substrate processing. In the processive mode, Dicer is dicing long dsRNA molecules in a set of consecutive dicing where ATP‐dependent activity of the helicase domain “feeds” the substrate into Dicer. In the distributive mode, Dicer performs a single cleavage and then binds a new substrate. (C) Variability of lengths of pre‐miRNAs in human, *C. elegans*, *D. melanogaster* and *A. thaliana*. Pre‐miRNA lengths were estimated for miRNAs having both mature miRNA strands annotated in the miRBase (Kozomara *et al*, 2019). The curve was smoothed by calculating average length for five adjacent miRNAs, points in the graph correspond to the central size of the five. (D) Several examples of murine mirtrons and miRNA stem‐loop miRNA precursors from the sponge *Amphimedon* and slime mold *Dictyostelium*. Stem‐loop structures were obtained from miRBase (Kozomara *et al*, 2019). Annotated mature miRNAs are highlighted in red font when on the ascending strand (5p miRNA) and blue font when on descending strand (3p miRNA).

Dicer substrates can be classified in different ways. Here, we divide substrates into three groups according to their structure and predictability of small RNA sequence (Fig 2A).

### Perfectly complementary long dsRNAs with a blunt end

These are typically produced by an RNA‐dependent RNA polymerase (RdRP) that synthesizes the antisense strand from the end of a template. It may be a hallmark of replicating viral RNA and its processing by Dicer one of the mechanisms of innate immunity (reviewed in Hur, 2019). RdRPs are also an intrinsic element in many RNA silencing mechanisms (e.g., in transcriptional silencing in plants and *S. pombe*; Mourrain *et al*, 2000; Colmenares *et al*, 2007) where RdRPs convert specific RNAs into Dicer substrates. A blunt‐end dsRNA can be recognized through the PAZ domain (Zhang *et al*, 2002; MacRae *et al*, 2007). However, the point‐of‐entry for long dsRNA into Dicer may also be the RIG‐I‐like N‐terminal helicase domain (Welker *et al*, 2011; Sinha *et al*, 2018). In the processive mode of dicing (Fig 2B), the ATPase activity of the helicase domain facilitates the enzyme's movement along the substrate, which is processed by a single Dicer molecule in an ATP‐dependent series of consecutive dicing (Zamore *et al*, 2000; Bernstein *et al*, 2001; Ketting *et al*, 2001; Nykanen *et al*, 2001; Cenik *et al*, 2011; Naganuma *et al*, 2021).

### Perfectly and nearly perfectly complementary long dsRNAs with longer single‐stranded overhangs

Such dsRNAs typically arise from genomic transcription followed by intra or intermolecular base pairing of cellular transcripts (reviewed in Chen & Hur, 2022). Such structures usually have one or two long single‐stranded overhangs (Fig 2A), as such transcripts are unlikely to match precisely to generate a blunt end. This precludes direct access of Dicer to ends of the double‐stranded region. However, Dicer is able to dice such substrates by a poorly understood endonucleolytic activity (Zhang *et al*, 2002), which yields fragments with two nucleotide overhangs, which are then efficiently recognized and processed by Dicer.

### Small hairpin substrates

Small hairpin substrates are common substrates for the biogenesis of miRNA‐like molecules. Annotated miRNAs in animals, plants, and other taxonomic groups (Kozomara *et al*, 2019) show that miRNAs are produced from a heterogeneous assortment of substrates that are not processed in a uniform way. At the same time, a common feature of miRNAs is that their biogenesis results in a small RNA with a defined sequence.

In plants, miRNAs are generated by a single Dicer, DCL‐1, that cuts a miRNA precursor twice. Many plant miRNAs evolved from eroded larger inverted repeats originally producing long dsRNA (reviewed in Svoboda & Di Cara, 2006) and often suppress complementary sequences by RNAi‐like endonucleolytic cleavage. Consequently, many plant miRNAs retain longer stems (Fig 2C) and are diced by DCL1 in two consecutive cleavages in an ATP‐dependent manner reminiscent of siRNA biogenesis (Fig 2B).

Canonical animal miRNAs are a distinct small RNA class whose biogenesis involves a two‐step processing by two RNase III family proteins—Drosha and Dicer (Fig 2B). Nuclear Drosha generates pre‐miRNA, a small precursor hairpin, from a long pri‐miRNA transcript. Subsequently, cytoplasmic Dicer cleaves the pre‐miRNA and releases a ~ 22 nt miRNA, which is loaded onto Argonaute. Canonical miRNAs in Eumetazoan animals typically emerge *de novo* (Chen & Rajewsky, 2007; Meunier *et al*, 2013), presumably from random small hairpin structures entering the miRNA biogenesis machinery. Given the distinct modes of biogenesis and independent adaptation of animal Dicers for miRNA biogenesis, miRNAs in Eumetazoan animals have shorter stems and smaller loops than plant miRNAs while showing slight differences in precursor length distribution (Fig 2C). There are also noncanonical animal miRNAs, such as mirtrons, which are Drosha‐independent, and are Dicer hairpin substrates derived from specific small spliced‐out introns (Fig 2D). How the biogenesis of annotated miRNAs from species from other taxonomic groups (e.g., *Dictyostelium* or *Phytophthora*) occurs is unclear. But the precursors of these miRNAs seem to have longer stems and loops, such as plant precursors do (Fig 2D).

Taken together, Dicer substrates have different structural features, which facilitate their recognition and cleavage. Three types of endonucleolytic cleavages by Dicer can be recognized: ATP‐dependent processive cleavage of dsRNA from its terminus, poorly understood internal endonucleolytic cleavage of long dsRNA with inaccessible ends, and loop removal from small hairpin miRNA precursors.

## Dicer Function at the Molecular Level

While Dicer was identified as an enzyme producing small RNAs more than two decades ago (Bernstein *et al*, 2001), only recent structural analyses of Dicers in plants (Wang *et al*, 2021; Wei *et al*, 2021) *Drosophila* (Jouravleva *et al*, 2022; Su *et al*, 2022; Yamaguchi *et al*, 2022) and mammals (Liu *et al*, 2018; Zapletal *et al*, 2022; Lee *et al*, 2023b) have provided detailed structural insights into miRNA and siRNA biogenesis by Dicer. Several Dicer homologs were captured in apo form and in multiple RNA‐bound states (Table 1), revealing details about substrate selection, catalysis, and product release, and how Dicer cooperates with dsRBP cofactors during RNA processing. These structural data from multiple organisms allow us to take a closer look at common and derived features of Dicer in substrate recognition and processing.

**Table 1:** Available structures of full‐length Dicers.

PDB ID	Protein/complex designation	Species	Note	References
2FFL	Dicer	*Giardia*	Apo form	MacRae *et al* (2006)
2QVW	Dicer	*Giardia*	Apo form	MacRae & Doudna (2007)
7ELD	DCL1–pri‐miRNA complex	*Arabidopsis*	No Mg/ATP, pri‐miRNA dicing state	Wei *et al* (2021)
7ELE	DCL1–pre‐miRNA complex	*Arabidopsis*	No Mg/ATP, pre‐miRNA dicing state	Wei *et al* (2021)
7VG3	DCL‐3–30 bp RNA complex	*Arabidopsis*	Dicing state	Wang *et al* (2021)
7VG2	DCL‐3–40 bp RNA complex	*Arabidopsis*	Dicing state	Wang *et al* (2021)
6BUA	Dcr‐2 cap‐core	*Drosophila*	Apo form	Sinha *et al* (2018)
7V6B	Dcr‐2:R2D2	*Drosophila*	Apo form	Yamaguchi *et al* (2022)
7V6C	Dcr‐2:R2D2:siRNA	*Drosophila*	Composite recognition/strand select	Yamaguchi *et al* (2022)
7W0A	Dcr‐2:LOQS‐PD	*Drosophila*	Dimer	Su *et al* (2022)
7W0B	Dcr‐2:LOQS‐PD:50 bp dsRNA	*Drosophila*	−ATP, apo state	Su *et al* (2022)
7W0C	Dcr‐2:LOQS‐PD:50 bp dsRNA	*Drosophila*	+ATP, Mg^2+^, early‐translocation	Su *et al* (2022)
7W0D	Dcr‐2:LOQS‐PD:50 bp dsRNA	*Drosophila*	+ATP, Mg^2+^, mid‐translocation	Su *et al* (2022)
7W0E	Dcr‐2:LOQS‐PD:50 bp dsRNA	*Drosophila*	+ATP, Mg^2+^, dicing	Su *et al* (2022)
7W0F	Dcr‐2:LOQS‐PD:50 bp dsRNA	*Drosophila*	+ATP, Mg^2+^, post‐dicing	Su *et al* (2022)
8DGI	Dcr‐1:LOQS‐PB	*Drosophila*	Apo form, closed conformation (Ia)	Jouravleva *et al* (2022)
8DGJ	Dcr‐1:LOQS‐PB	*Drosophila*	Apo form, closed conformation (Ib)	Jouravleva *et al* (2022)
8DFV	Dcr‐1:LOQS‐PB:pre‐let‐7	*Drosophila*	Ca^2+^, dicing‐competent state (IIa)	Jouravleva *et al* (2022)
8DG5	Dcr‐1:LOQS‐PB:pre‐let‐7	*Drosophila*	Mg^2+^, dicing‐competent state (IIb)	Jouravleva *et al* (2022)
8DG7	Dcr‐1:LOQS‐PB:pre‐let‐7	*Drosophila*	Mg^2+^, 5′ arm dicing state (III)	Jouravleva *et al* (2022)
8DGA	Dcr‐1:LOQS‐PB:pre‐let‐7	*Drosophila*	Mg^2+^, 3′ arm dicing state (IV)	Jouravleva *et al* (2022)
7YZ4	Dicer	Mouse	Apo form	Zapletal *et al* (2022)
7YYM	Dicer:pre‐miR‐15	Mouse	Pre‐dicing state	Zapletal *et al* (2022)
7YYN	DicerO:pre‐miR‐15	Mouse	Dicing state	Zapletal *et al* (2022)
7ZPK	Dicer:pre‐miR‐15:TARBP2	Mouse	Pre‐dicing state	Zapletal *et al* (2022)
7ZPI	Dicer:pre‐miR‐15:TARBP2	Mouse	Dicing state	Zapletal *et al* (2022)
5ZAK	Dicer:TARBP2	Human	Apo form	Liu *et al* (2018)
5ZAL	Dicer:TRBP:pre‐let‐7	Human	Pre‐dicing (class I)	Liu *et al* (2018)
5ZAM	Dicer:TRBP:pre‐let‐7	Human	Pre‐dicing (classII)	Liu *et al* (2018)
7XW2	Dicer‐pre‐miRNA	Human	Dicing state	Lee *et al* (2023b)
7XW3	Dicer	Human	Apo form	Lee *et al* (2023b)

### Dicer helicases determine how the enzyme selects and loads its substrate

The determined structures of Dicer from invertebrates (*Drosophila* Dicer‐1 and Dicer‐2) and Dicer‐1 from mammals all adopt very similar closed “L”‐shaped conformations in the apo state (Liu *et al*, 2018; Jouravleva *et al*, 2022; Su *et al*, 2022; Yamaguchi *et al*, 2022; Zapletal *et al*, 2022; Lee *et al*, 2023b). In this autoinhibited conformation reflecting *in vitro* data (Ma *et al*, 2008), the helicase domain is associated with RNase IIIb and limits the loading of RNA into the catalytic site. The helicase domains of these Dicers have diverged during evolution. For example, *Drosophila* Dicer‐1 has a degenerate helicase domain and is an ATP‐independent enzyme (Tsutsumi *et al*, 2011), *Drosophila* Dicer‐2 has a conserved helicase domain that hydrolyzes ATP (Zamore *et al*, 2000; Nykanen *et al*, 2001; Cenik *et al*, 2011; Sinha *et al*, 2015), and mammalian Dicer‐1, despite conservation of its helicase domain, does not hydrolyze ATP (Zhang *et al*, 2002). Accordingly, Dicers employ distinct mechanisms of substrate recognition and loading. This view is in line with the recent attempt to reconstruct ancestral helicase sequences of animal Dicers and analyze their properties *in vitro* (Aderounmu *et al*, 2023).

**Figure 3:**
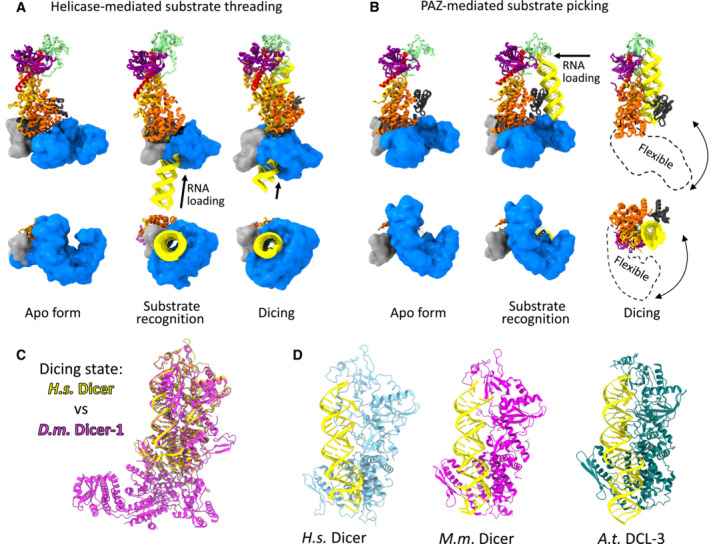
Substrate recognition and dicing by Dicer (A) Substrate recognition and threading of dsRNA substrate through the helicase domain exemplified on *Drosophila* Dicer‐2 (PDB IDs: 7W0B, 7W0A [monomer], and 7W0E) (Su *et al*, 2022). (B) Substrate recognition involving PAZ domain exemplified by miRNA recognition by mouse Dicer‐1 (PDB ID: 7YZ4, 7YYM, and 7YYN) (Zapletal *et al*, 2022). In the dicing state, the helicase becomes flexible and its precise position cannot be determined. (C) Overlay of the dicing state of human *Dicer‐1* (in yellow) and *Drosophila* Dicer–1 (in magenta; PDB IDs: 7XW2 and 8DG7) (Jouravleva *et al*, 2022; Lee *et al*, 2023b). (D) Dicing states of human, murine and *Arabidopsis* Dicers (PDB IDs: 7XW2, 7YYN, and 7VG2; Wang *et al*, 2021; Zapletal *et al*, 2022; Lee *et al*, 2023b). Position of the helicase domain in these dicing states was not determined.

Upon RNA binding, the horseshoe‐shaped structure of the helicase domain of *Drosophila* Dicer‐2 associates with the DUF283 domain to form a ring (similar to RIG‐I‐like receptor) that threads the RNA substrate into the enzyme (Fig 3A; Sinha *et al*, 2018; Su *et al*, 2022). Using this mechanism, Dicer‐2 can process not only dsRNA with a blunt end (Sinha *et al*, 2018) but also those with an overhang at the 3′‐end, as suggested by quantitative biochemical evidence (Cenik *et al*, 2011), single‐molecule fluorescent microscopy (Naganuma *et al*, 2021), and a recent structural study (Su *et al*, 2022). While it is still controversial whether or not the initial binding of RNA requires ATP, the subsequent translocation of the RNA substrate through the helicase‐DUF283 ring is ATP‐dependent (Sinha *et al*, 2018; Naganuma *et al*, 2021; Singh *et al*, 2021; Su *et al*, 2022).

By contrast, the helicase domain of mammalian Dicer‐1 is not rearranged and remains in the closed “L”‐shaped conformation and encircles the pre‐miRNA substrate during initial RNA binding. This mechanism discriminates between authentic miRNA precursors and other RNA hairpins by anchoring three elements: the RNA ends, the central region, and the terminal loop (Fig 3B). Upon binding of the substrate (and TARBP2), Dicer switches into an active open state that allows repositioning of the substrate into the RNA processing center. This substrate repositioning requires disengagement of the helicase domain and DUF283 from the Dicer core, and the unlocked helicase becomes highly flexible in the dicing state (invisible in cryo‐EM), and the same has been reported for Dicer in mice (Zapletal *et al*, 2022) and humans (Lee *et al*, 2023b). Interestingly, the helicase domain of Dicer‐1 in flies remains fully bound to the Dicer core and undergoes only a small conformational change toward an open conformation that is sufficient to accommodate authentic miRNA precursors (Jouravleva *et al*, 2022).

Structural studies in plants showed that DCL‐3, an siRNA‐producing enzyme, adopts the same active conformation as mammalian miRNA‐producing Dicer‐1, whose helicase domain and DUF283 are disengaged from the Dicer core and are highly flexible (Wang *et al*, 2021). In contrast, the miRNA‐producing DCL‐1 in plants, which combines the activities of both Drosha and Dicer in mammals, uses a helicase‐mediated threading mechanism typical of the siRNA‐producing Dicers in animals. The DCL‐1 structures showed that the helicase‐DUF238 threading ring transfers the RNA substrate in an ATP‐dependent manner, while the enzyme performs two cuts, first pri‐miRNA to form pre‐miRNA and then pre‐miRNA to miRNA (Wei *et al*, 2021).

### Dicing mechanism

The set of recent structures showed that the adaptation of Dicer to specific roles in different organisms and pathways (Box 1) is encoded in the substrate selection and in the loading mechanism, while the actual mechanism of catalysis remains invariant. The superposition of Dicer–RNA structures, regardless of organism or pathway, shows virtually identical conformations of the core elements involved in the catalysis (Fig 3C and D; Wang *et al*, 2021, Jouravleva *et al*, 2022 #744; Zapletal *et al*, 2022; Lee *et al*, 2023b). This includes the insertion of 3′ and 5′ ends of pre‐miRNA into a basic pocket of the PAZ‐Platform cassette, which aligns the substrate and determines the dicing site. The pre‐miRNA is further aligned in the positively charged groove formed by the RNase IIIa/b domains. The internal dsRBD of Dicer clamps the RNA in the catalytic sites of Dicer. A recent study proposed that the dsRBD of human Dicer recognizes the GYM motif (G guanine; Y, paired C/U; M, mismatched nucleotide) and stabilizes the interaction between the RNA and Dicer for a more efficient dicing reaction (Lee *et al*, 2023b). This molecular interaction could explain the evolutionary conservation of the GYM motif in a subset of miRNAs (Lee *et al*, 2023a).

Box 1 **Characteristics of Dicer structures**

***Drosophila* Dicer‐2**

*Inactive form*: L‐shaped.

*Active form*: the horseshoe‐shaped structure of the helicase domain associates with the DUF283 domain to form a ring that threads the RNA substrate into the enzyme (yet side‐loading (ATP‐independent; distributive) can occur for the 3′overhang substrate).

***Drosophila* Dicer‐1**

*Inactive form*: L‐shaped.

*Active form*: small opening of the helicase domain during the miRNA substrate loading, the helicase stays associated with the core.

**Vertebrate Dicer‐1**

*Inactive form*: L‐shaped.

*Active form*: the helicase domain and DUF283 entirely disengage from the Dicer core.

**Plant DCL‐3**

*Inactive form*: not published.

*Active form*: the helicase domain and DUF283 entirely disengage from the Dicer core.

**Plant DCL‐1**

*Inactive form*: not published.

*Active form*: the horseshoe‐shaped structure of the helicase domain associates with the DUF283 domain to form a ring that threads the RNA substrate into the enzyme.

### Internal and external dsRBDs modulate Dicer's activities

**Figure 4:**
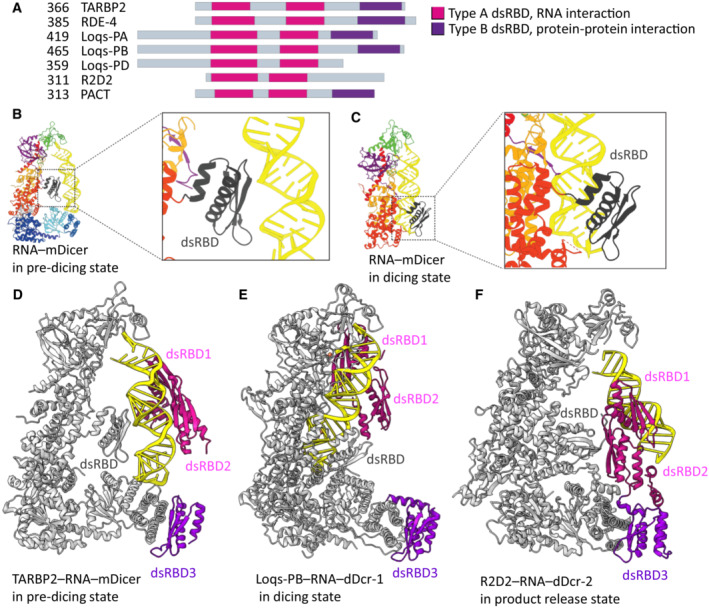
Different modes of RNA binding by dsRBD in substrate selection, dicing, and substrate release by Dicer (A) Overview of Dicer cofactors that contain dsRBD. (B) In pre‐dicing state, the internal dsRBD of mouse Dicer unusually uses its β‐sheet surface to recognize RNA (Zapletal *et al*, 2022; PDB ID: 7YYM). (C) In dicing state, the internal dsRBD of mouse Dicer utilizes its α‐helical face for RNA recognition (Zapletal *et al*, 2022; PDB ID: 7YYN). (D) TABRB2 dsRBD1 and 2 bind the central region of pre‐miRNA (Zapletal *et al*, 2022; PDB ID: 7ZPK). (E) Dicer‐1 dsRBD and Loqs‐PB dsRBD encircle the RNA and lock it in the catalytic site (Jouravleva *et al*, 2022; PDB ID: 8DFV). (F) While Dicer–2 releases the substrate, R2D2 asymmetrically recognizes the end of the siRNA duplex with the higher base‐pairing stability (PDB ID: 7V6C). dDcr‐1, *Drosophila* Dicer–1; dDcr–2, *Drosophila* Dicer–2; mDicer, murine Dicer1.

Recent structures of mammalian Dicer‐1 unexpectedly revealed that its internal C‐terminal dsRBD has two distinct RNA‐binding sites. The first RNA‐binding site, situated on the β‐sheet surface of the domain, is responsible for binding the central double‐helical region of the pre‐miRNA in the pre‐dicing state. In contrast, the second RNA‐binding site is located on the α‐helical surface that is at the opposite side of the domain and is used to clamp RNA in the catalytic sites of Dicer in the dicing state (Fig 4B and C; Zapletal *et al*, 2022; Lee *et al*, 2023b). The fact that the dsRBD switches between its two RNA‐binding sites during the multistep catalysis process of Dicer demonstrates the remarkable plasticity of this important domain. The structural studies of *D.m*. Dicer‐2 showed that the dsRBD also helps to induce bending of the dsRNA substrate, which facilitates the proper alignment of the substrate with the core of Dicer and its translocation into the PAZ domain (Su *et al*, 2022).

Additionally, Dicers associate with accessory dsRBD‐containing proteins such as TARBP2, ADAR1, PKR, PACT in mammals, and Loquacious‐PA/PB/PD and R2D2, in invertebrates (Fig 4A; Hansen *et al*, 2019). These external regulatory dsRBD‐containing proteins associate with the Dicer helicase domain via their C‐terminal Type B dsRBD (protein–protein interacting domain; Doyle & Jantsch, 2002; Hansen *et al*, 2019) as in TARPB2, R2D2, and Loquacious‐PA/PB (Fig 4D–F) or via unstructured regions as in Loquacious‐PD (Su *et al*, 2022). In general, these binding partners facilitate miRNA or siRNA precursor interaction with Dicers. Recent structural work on *D.m*. Dicer‐2 showed that the C‐terminal tail of Loquacious‐PD binds to the helicase domain of Dicer‐2 and its two dsRBDs support substrate binding (Su *et al*, 2022). However, the dsRBDs of Loquacious‐PD were not observed in the density map of the initial binding state, suggesting that the dsRBDs have only an assisting role in the initial binding of dsRNA to the Dicer‐2 helicase domain. In mammals, TARBP2 stimulates the transition from the pre‐dicing to dicing‐competent state (Zapletal *et al*, 2022). In the pre‐dicing state, the dsRBD3 of TARBP2 stably associates with the Dicer‐1 helicase (Fig 4D; Liu *et al*, 2018; Zapletal *et al*, 2022), while the first two dsRBDs bind dsRNA in a flexible manner, similarly as observed for Loquacious‐PD (Fig 4D; Zapletal *et al*, 2022). Akin to TARBP2, the C‐terminal dsRBD3 of Loquacious‐PB interacts with the helicase domain of *D.m*. Dicer‐1. By contrast, the first two dsRBDs of Loquacious‐PB are stably bound to the miRNA precursor and assist *D.m*. Dicer‐1 in the positioning of the pre‐miRNA into the RNA processing center (Fig 4E; Jouravleva *et al*, 2022).

Recent structures of the Dicer‐2‐R2D2‐siRNA complexes provided insight into substrate release and strand‐selection state mediated by R2D2 in the siRNA‐loading process. In this state, the internal dsRBD of Dicer is dissociated from the siRNA and R2D2 stably and asymmetrically recognizes the end of the siRNA duplex with the higher base‐pairing stability, while the other end (the guide strand for target silencing) is accessible for loading onto AGO2 (Fig 4F; Yamaguchi *et al*, 2022).

In previous structural studies, the isolated dsRBDs were shown to bind dsRNA with little or no specificity (Green & Mathews, 1992; St Johnston *et al*, 1992; Bass *et al*, 1994; Bycroft *et al*, 1995; Kharrat *et al*, 1995; Nanduri *et al*, 1998; Ryter & Schultz, 1998; Lehmann & Bass, 1999; Fierro‐Monti & Mathews, 2000; Ramos *et al*, 2000; Blaszczyk *et al*, 2004; Gan *et al*, 2006; Stefl *et al*, 2006; Masliah *et al*, 2018), but several studies have reported that they can bind preferentially when irregularities (e.g., mismatches, internal loops, and stem‐loop junctions) are present in the RNA double helix (Stefl *et al*, 2005, 2010; Wang *et al*, 2011; Jayachandran *et al*, 2016; Lazzaretti *et al*, 2018; Yadav *et al*, 2020). It was shown that the irregularities widen the minor groove of the RNA double helix, thereby facilitating the positioning of the α1 helix of the dsRBD on dsRNA. Recent Dicer–RNA structures with dsRBD‐containing regulatory proteins show that the mode of dsRBD binding is governed by the dynamic structural context of multistep Dicer catalysis. This versatility of dsRBDs ranges from nonspecific interactions with multiple dsRNA registers during initial substrate recruitment to highly specific interactions (mainly dsRBDs α1 helix in the minor dsRNA groove) that directly modulate Dicer catalysis and control substrate release to properly feed AGO2 with a guide strand for target silencing (Liu *et al*, 2018; Jouravleva *et al*, 2022; Su *et al*, 2022; Yamaguchi *et al*, 2022; Zapletal *et al*, 2022; Lee *et al*, 2023b).

## Ancestral and Derived Dicer Functions

For a more detailed overview of the evolution of Dicer and its paralogs, we refer readers to the works of Mukherjee *et al* (2013) and Jia *et al* (2017). Here, we will discuss only selected issues, which have emerged from the recent structural analyses. As mentioned earlier, the complex canonical Dicer architecture found in animals, plants, fungi, and Protista implies that the canonical Dicer architecture with a functional ATPase/helicase had evolved already in the common ancestor of all three multicellular kingdoms and at least some extant Protista. The RIG‐I‐like helicases serve as cytoplasmic dsRNA sensors for antiviral immunity across animals (Yoneyama & Fujita, 2007; Kowalinski *et al*, 2011; Guo *et al*, 2013). RIG‐I recognizes blunt‐end dsRNA with 5′ triphosphate (Kowalinski *et al*, 2011), which is a hallmark of viral replication. The primary function of the canonical Dicer architecture was thus most likely linked to dsRNA processing in antiviral defense in early eukaryotes living in aquatic environments which hosted virioplankton. While it is unknown how dense and diverse virioplankton was in primeval oceans, the exposure of early eukaryotes to viruses was likely intense. This notion is consistent with high throughput analyses of seawater nowadays, which reveal unexpected density and diversity of extant virioplankton, including RNA viruses (Vlok *et al*, 2019; Wolf *et al*, 2020). The antiviral role is highly plausible for explaining the emergence and success of the canonical Dicer architecture. Given the major regulatory potential of sequence‐specific targeting by small RNA, it is not surprising that other RNA silencing pathways emerged, such as the gene expression‐regulating miRNA pathway or transcriptional silencing mechanisms.

The major requirement for the miRNA pathway is biogenesis of precisely defined small RNAs, which enables their specific engagement in gene regulation. In plants and animals, miRNA biogenesis involves distinct mechanisms (reviewed in Rogers & Chen, 2013; Bartel, 2018). In plants, two cleavages by DCL1 release a miRNA from its precursor. In animals, the mechanism involves first nuclear cleavage by Drosha and then cytoplasmic cleavage by Dicer. In this context, Drosha appears as a highly specialized animal Dicer variant emerging from Dicer duplication early in Metazoan evolution. Animal Dicer‐1 shows distinct adaptations to miRNA biogenesis in different animal lineages further supporting the notion that animal miRNA biogenesis by Dicer is a derived character. The main adaptation concerned the helicase domain, whose evolution in animal Dicers took diverse paths. In *C. elegans* DCR‐1, the helicase is fully functional as the enzyme is efficiently processing both types of substrates—long dsRNA and pre‐miRNAs. In *Drosophila*, the helicase of miRNA‐producing Dicer‐1 lost its original function and degenerated, while siRNA‐producing Dicer‐2 carries a functional RIG‐I‐like helicase hydrolyzing ATP and feeding dsRNA substrate into the enzyme. A recent attempt to reconstruct ancestral helicase sequences of animal Dicers suggests that decline of the functionality of the helicase started relatively early in the lineage leading to Deuterostomes and vertebrates (Aderounmu *et al*, 2023). Notably, Dicer in the earliest branching animal groups (ctenophores, sponges, and cnidarians; Schultz *et al*, 2023) uses a binding partner, which resembles HYL1 from plants rather than Loquacious/TARBP2 (Moran *et al*, 2013) suggesting that some miRNA‐like mechanism preceded the existence of miRNA pathways in animals.

Mammalian Dicer‐1, which primarily generates miRNAs, represents a remarkable case where the helicase lost its helicase/ATPase function, yet amino acid residues critical for ATPase function are highly conserved across mammals. Structural and functional analyses of the helicase showed that it acquired an ATP‐independent unique structural role in miRNA biogenesis (Zapletal *et al*, 2022). The previously observed dicing‐incompetent “pre‐dicing state” where a pre‐miRNA interacts with PAZ, dsRBD, and helicase domains (Liu *et al*, 2018) appears to be a specific adaptation of mammalian Dicer for recognition of bona fide pre‐miRNAs. In *Drosophila*, the single‐stranded region of the pre‐miRNA also interacts with the helicase but the arrangement is different from that in mammals (Jouravleva *et al*, 2022).

As mentioned above, a recent reconstruction of ancestral helicases of animal Dicers (Aderounmu *et al*, 2023) suggests that degeneration of the helicase activity in the lineage leading to mammals likely started already in early deuterostomes and the helicase was inactive in vertebrate ancestors already. This creates a remarkable paradox—removal of HEL1 from a mammalian Dicer increases its ability to dice long dsRNA (Ma *et al*, 2008; Flemr *et al*, 2013), which is the opposite of its original role to facilitate long dsRNA dicing. Furthermore, there is no evidence that mammalian Dicer lacking HEL1 is truly processive. Analyses of small RNA populations generated by the full‐length Dicer and the shorter isoform suggest that the shorter isoform might only be more active (Flemr *et al*, 2013; Zapletal *et al*, 2022).

## Summary and Outlook

Structural analyses of Dicers with canonical Dicer architectures reveal features associated with miRNA and siRNA biogenesis and provide a good framework for interpreting future structures and/or their predictions (Box 2). It is likely that future studies of Dicer in nematodes, mollusks and other animals will identify additional unique adaptations of Dicer for miRNA biogenesis. At the same time, some organisms rely on Dicers, which retain only the core module and the mechanism by which these Dicers generate small RNAs of defined length has not yet been determined. AlphaFold‐based models (Varadi *et al*, 2022) offer interesting insights into predicted structural organization of these divergent Dicers and suggest that the future may hold additional mechanisms for small RNA biogenesis by Dicer.

Box 2 **In need of answers**

How do noncanonical Dicers, such as DrnA and DrnB in *Dictyostelium*, recognize and cleave substrates to produce defined lengths of small RNAs? Are there other Dicer architectures in Protists?

A single Dicer in *C. elegans* produces both, miRNAs and siRNAs. Are long dsRNA and pre‐miRNA substrates recognized and cleaved in a similar way or differently? That is, is long dsRNA recognized and loaded into the enzyme through the helicase domain and do pre‐miRNAs interact with Dicer primarily through the PAZ domain? Cryo‐EM structures of *C. elegans* Dicer with both substrates would answer this question.

How does AGO bind Dicer and select 5p/3p miRNAs? It remains unclear how AGO interacts with Dicer and binds cleavage products in different model organisms. Once a cleavage product is released from Dicer, thermodynamic sensing promotes selection and loading of the main strand. However, structural insights into the organization of the RISC loading complex and the loading process are still needed.

What is the physiological relevance of PACT, a mammalian TARBP2 paralog? Is it having any specific role?

How do other Dicer‐binding partners regulate its activity/substrate specificity?

## Author contributions

**David Zapletal:** Visualization; writing – review and editing. **Karel Kubicek:** Visualization; writing – review and editing. **Richard Stefl:** Conceptualization; funding acquisition; visualization; writing – original draft; writing – review and editing. **Petr Svoboda:** Conceptualization; funding acquisition; visualization; writing – original draft; writing – review and editing.

## Disclosure and competing interests statement

The authors declare that they have no conflict of interest.
